# Study on the Effect of Hot and Humid Environmental Factors on the Mechanical Properties of Asphalt Concrete

**DOI:** 10.3390/ma17204942

**Published:** 2024-10-10

**Authors:** Xin Yan, Zhigang Zhou, Yingjia Fang, Chongsen Ma, Guangtao Yu

**Affiliations:** 1 School of Traffic & Transportation Engineering, Changsha University of Science and Technology, Changsha 410114, China; yanxin@stu.csust.edu.cn (X.Y.); zhou_zgcs@163.com (Z.Z.); fangyinjia11@163.com (Y.F.); machongsen@stu.csust.edu.cn (C.M.); 2 National Key Laboratory of Green and Long-Life Road Engineering in Extreme Environment, Changsha University of Science and Technology, Changsha 410114, China

**Keywords:** asphalt concrete, dynamic modulus, environmental factors, thermo-oxidative aging, dry–wet cycle, uniaxial compression test, master curve

## Abstract

To investigate the effect of hot and humid environmental factors on the mechanical properties of asphalt mixtures research, in this paper, the dynamic modulus of asphalt mixtures under the effects of aging, dry–wet cycling, and coupled effects of aging and dry–wet cycling were measured by the simple performance tester (SPT) system, and the dynamic modulus principal curves were fitted based on the sigmoidal function. The results show that under the aging effect, the dynamic modulus of asphalt mixture increases with the aging degree; the dynamic modulus of short-term aged, medium-term aged, long-term aged, and ultra-long-term aged asphalt mixtures increased by 9.3%, 26.4%, 44.8%, and 57%, respectively, compared to unaged asphalt mixtures at 20 °C and 10 Hz; the high-temperature stability performance is enhanced, and the low temperature cracking resistance performance is enhanced; under the dry–wet cycle, the aging effect of asphalt water is more obvious in the early stage, and dynamic modulus of resilience of the mixture is slightly increased. In the long-term wet–dry cycle process, water on the asphalt and aggregate erosion increased, the structural bearing capacity attenuation, and the dynamic modulus of rebound greatly reduced at 20 °C and 10 Hz. For example, the dynamic modulus of asphalt mixtures with seven wet and dry cycles increased by 3% compared to asphalt mixtures without wet and dry cycles, and the dynamic modulus of asphalt mixtures with 14 cycles of wet and dry cycles and 21 cycles of wet and dry cycles decreased by 10.8% and 16.5%, respectively, compared to asphalt mixtures without wet and dry cycles. The main curve as a whole shifted downward; the high-temperature performance decreased significantly; in the aging wet–dry cycle coupling, the aging asphalt mixture is more susceptible to water erosion, and the first wet–dry cycle after the mix by the degree of water erosion is relatively small, along with the dynamic modulus of rebound. The dynamic modulus of resilience is relatively larger, and the high-temperature performance is relatively better, while the low-temperature performance is worse.

## 1. Introduction

Looking back on several decades of transportation infrastructure development, China heavily relied on asphalt pavement structures in the early stages of constructing its highways and urban roads. However, over time, these roads have experienced varying degrees of damage after being in service for different durations. This deterioration has led to a decline in their functionality, making them inadequate to meet the continuously increasing traffic demands. Consequently, there is a pressing need for maintenance and renovation to address these issues [1,2,3]. In the process of maintenance and transformation, by which in the hot and humid areas, there are many times on the old concrete pavement with asphalt pavement. For different periods of paving asphalt surface layer, has experienced different years of wet and hot aging, asphalt concrete resistance to deformation, and bearing capacity by different degrees of decay [4,5,6,7]. In the Design Code for Highway Asphalt Pavements (JTG D50-2017) [8], new pavement design indexes have been added, and a number of control indexes are used for structural design according to different types of base structures. However, for the original pavement structure with good service performance, in the old road reconstruction design, the pavement structure and road base of the old road are regarded as semi-infinite spatial bodies, and the equivalent resilience modulus of the top surface of the old road is used as the resilience modulus of the road base for the reinforcement of the pavement structure to carry out the design of the retrofitting structure. This paving structure design does not consider the existing pavement’s modulus and its materials after natural aging, which will inevitably affect the ability of the new pavement structure to resist deformation, leading to a reduction in the pavement’s service life [9,10]. A series of studies have been conducted on the dynamic properties of asphalt mixtures.

In China, Hui Chen [11] et al. simulated the water damage of asphalt mixtures by freeze–thaw cycles, and the dynamic modulus and the master curve before and after freeze–thaw were used as the basis for evaluation. After the freeze–thaw cycle, the freeze–thaw modulus ratios all decreased with increasing temperature and decreasing frequency, i.e., it was concluded that the adverse effects of water damage on viscoelastic properties were manifested to a greater extent under high-temperature and low-frequency conditions. Rongxin Guo [12] et al. simulated the erosion of water on steel slag asphalt mixtures by means of dry–wet cycling of high-temperature hydrostatic water, investigated the change of dynamic modulus of the mixtures by the number of dry–wet cycling, and analyzed the mechanism of the performance decay by microscopic techniques. The study showed that the steel slag asphalt mixture after water erosion caused local cracking due to the hydration and expansion of steel slag, and the water intrusion led to a decrease in the stability of the overall structure, which caused the obvious decay of the dynamic modulus. Qingyi Xiao [13] et al. explored the feasibility of replacing aggregates with solid waste iron tailings and used conventional asphalt mixtures as a control to study the viscoelastic properties of solid waste asphalt mixtures and establish their master curves. Compared with conventional asphalt mixtures, the modulus of iron tailings mixtures is lower at low temperatures and slightly higher at high temperatures, and the modulus difference at room temperature is not significant, which indicates that their high-temperature stability and low-temperature crack resistance are slightly better.

Abroad, K. Al-Adham [14] et al. modified asphalt using different dosages of SBS and rubber to perform dynamic modulus tests and establish the corresponding master curves. Comparing the master curves under the same modifier, the high-dosage modified asphalt was always higher than the low-dosage modified asphalt, where the 6% SBS modified asphalt mix was at the highest throughout the frequency course. In the low-frequency range, there is a significant difference in the modulus of the mixes, and the modulus does not vary much in the high-frequency range. D. Han [15] et al. used SPT and M2F trapezoidal beam tests to compare the dynamic modulus of asphalt mixtures under two different stresses, bending and compression shear, and established strain-dependent models for the dynamic modulus of AC13-SBS and AC20-SBS based on the Bolzmann function model. It is shown that the strain dependence is highly sensitive to the test temperature, which is not prominent at low temperatures. When the temperature is higher, the strain dependence is more obvious due to the viscoelasticity of asphalt materials. The dynamic modulus in the bending and tensile modes decreases with increasing strain level and is “soft”, whereas the dynamic modulus in the compression and shear modes is the opposite and is “hard”. Md Rashadul lslam [16] et al. investigated the variability of dynamic modulus with different loading parameters (test frequency, load magnitude, and time between actions) and proposed more appropriate dynamic modulus test parameters, suggesting that a continuous loading pattern with a loading frequency of 10 Hz and a standard load of 0.7 MPa should be used. A.K. Arshad et al. [17] used nanosilica-modified asphalt to enhance the permeable asphalt mixture dynamic modulus and thus improve their rutting resistance.

In summary, scholars at home and abroad in the dynamic resilience modulus have carried out a lot of research work and achieved rich research results. However, there are still some limitations, mainly reflected in the following two aspects: in the study of the dynamic resilience modulus of asphalt concrete, scholars on the internal factors of the material, the external test conditions on the dynamic modulus of resilience of asphalt concrete to do a lot of research, and in the long-term complex and variable natural factors cyclic action of asphalt concrete dynamic resilience modulus change law research less, environmental factors simulation conditions single, did not consider the comprehensive impact of water erosion and aging. On the other hand, in the actual service process of asphalt concrete, due to the influence of specimen size, the indoor dynamic resilience modulus test method is limited, the dynamic resilience modulus of asphalt concrete core samples is less studied, and it is difficult to determine the value of dynamic resilience modulus of existing asphalt concrete. Therefore, for the old asphalt concrete pavement materials in hot and humid areas, through the indoor simulation of hot and humid environment, to carry out asphalt mixture in the hot and humid environment under the effect of erosion of the dynamic modulus of rebound change rule research, for the old pavement reconstruction and paving structural design of the old asphalt concrete design modulus of the basis.

The main innovations of this paper are as follows:

1. This paper mainly explores the change rule of dynamic modulus of return of asphalt mixtures under the combined effect of long-term complex and variable natural factors cycle.
2. Probes the first aging after the dry–wet cycle and the first dry–wet cycle after aging of different role orders on the dynamic modulus of resilience of the asphalt mixture change rule of law research.

## 2. Raw Materials and Gradation Design

### 2.1. Raw Material

In this paper, Shell Xinyue Foshan Asphalt Co., Ltd. (Foshan, China) was selected as the No. 70 matrix asphalt, and the results of its related technical indexes are shown in Table 1. Coarse aggregate was selected as pyrophyllite, and fine aggregate and mineral powder were selected as limestone; their related technical indexes are shown in Table 2, Table 3 and Table 4. The test results of the selected materials are in line with the “requirements of Technical Specification for Highway Asphalt Pavement Construction” (JTG E40-2004) [18].

### 2.2. Grading Design

**Figure 1:**
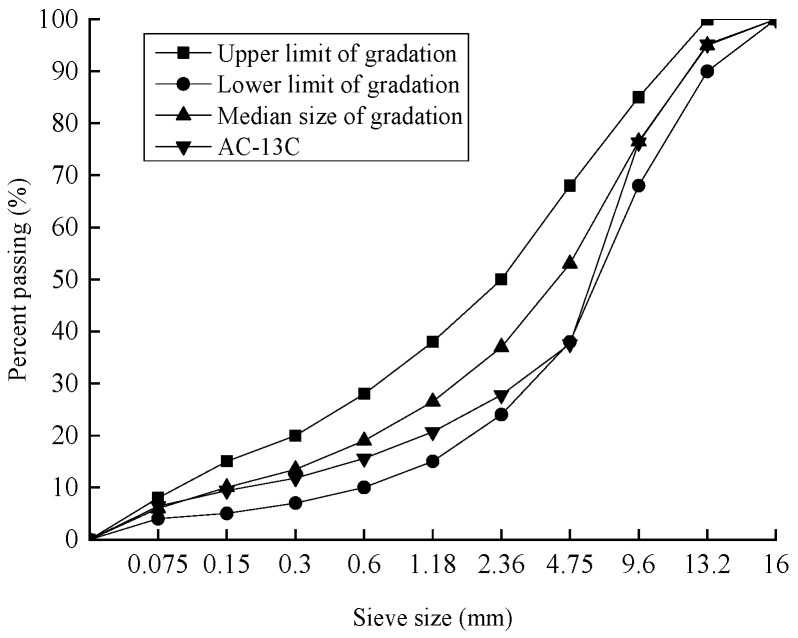
AC-13C grading curve.

In order to ensure consistency with the construction site and minimize the impact caused by the differentiation of materials between the indoor and construction sites, the target mix ratio was adopted as the gradation for the indoor simulation test through the investigation of the historical data on-site. The range of AC-13C gradation and the gradation curve are shown in Figure 1.

According to the above grading, the best oil-rock ratio of AC-13C is 4.8%, which is used to form the Marshall test specimen to obtain the volume parameters and Marshall test index of the specimen. At the same time, in order to verify whether its high-temperature stability, water stability performance is in line with the “Highway Asphalt Pavement Construction Technical Specification” (JTG F40-2017) [21] requirements, in accordance with the “Highway Engineering Asphalt and Asphalt Mixture Test Specification” (JTG E20-2011) [22] requirements for the rutting test, water-soaked Marshall test, freeze–thaw splitting test. Each test index is shown in Table 5 and Table 6, respectively, which are in line with the specification requirements.

## 3. Uniaxial Compression Dynamic Modulus Test

### 3.1. Specimen Preparation

According to the requirements of “Test Procedure for Asphalt and Bituminous Mixture in Highway Engineering” (JTG E20-2011), a cylindrical specimen with the size of Φ100 mm × 150 mm was obtained by compaction, core drilling, cutting and polishing using Superpave rotary compactor (Pine, New York, NY, USA), and core drilling machine (Foshan, China). The height and end face flatness of the specimens need to be measured before the test, and the error of the height of the specimens is ±2.5 mm, and the error of the end face flatness is less than 0.3 mm; otherwise, the samples should be discarded. Three parallel specimens were tested in each set of tests.

### 3.2. Test Protocol

**Figure 2:**
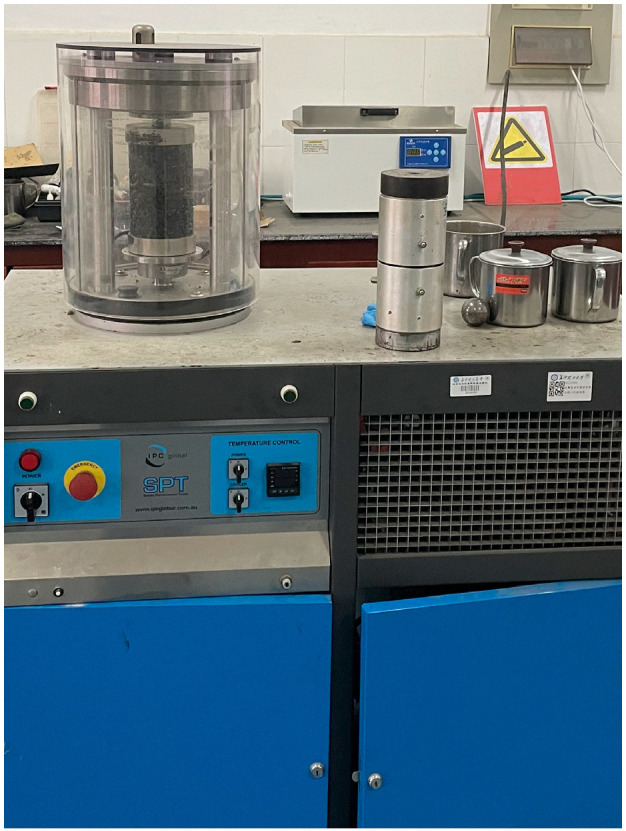
SPT test apparatus.

The temperature of the dynamic modulus test was selected to be 10 °C, 20 °C, 35 °C, and 50 °C, and the loading frequency was selected to be 0.1 Hz, 0.5 Hz, 1 Hz, 5 Hz, 10 Hz, and 20 Hz, all of which meet the requirements of the “Specification for the Design of Highway Asphalt Pavements” (JTG D50-2017). The dynamic modulus test was conducted using the SPT tester manufactured by IPC Australia, as shown in Figure 2. The test was applied as sinusoidal pressure with a load value of 13.5 kN, and the strain control method was adopted with a strain range of 85–115 με. The dynamic modulus was defined as shown in Equation:

(1) $$E_{\left(t\right)}=\left|\sigma_{\left(t\right)}\right|/\left|\varepsilon_{\left(t\right)}\right|=\sigma_{0}/\varepsilon_{0}$$

where $E_{\left(t\right)}$ is the dynamic resilience modulus; $\sigma_{\left(t\right)},\varepsilon_{\left(t\right)}$ are the dynamic stress and dynamic strain as a function of time; $\sigma_{0},\varepsilon_{0}$ are the amplitudes (modes) of dynamic stress and dynamic strain.

**Figure 3:**
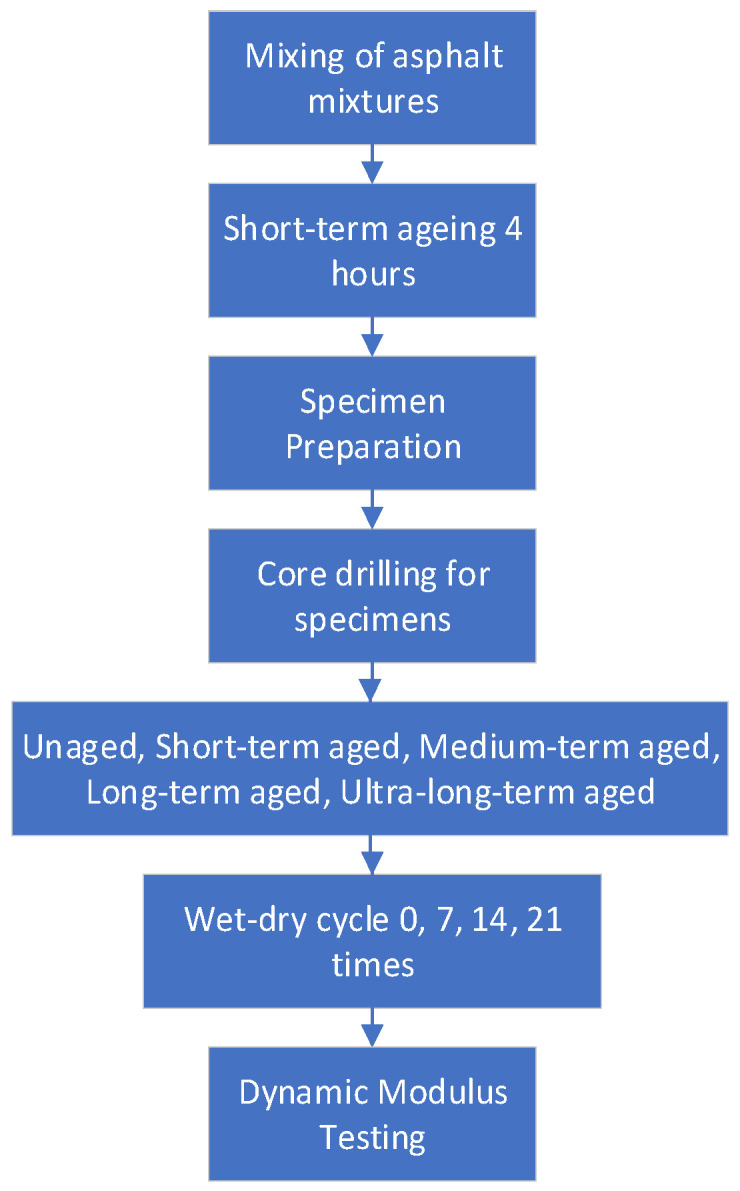
Test flow chart.

**Figure 4:**
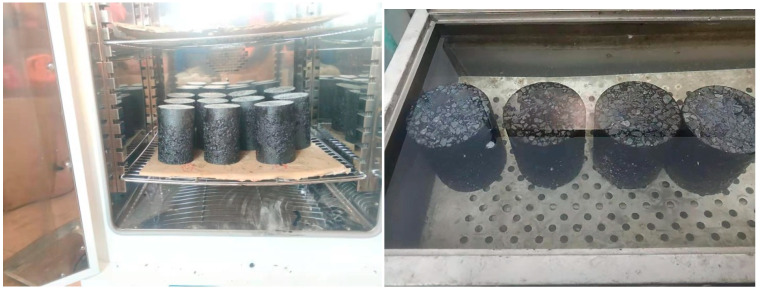
Aging test and dry–wet cycle.

As shown in Figure 3 and Figure 4, the aging test is selected from the oven heating method, the short-term aging test temperature is 135 ± 1 °C, and the long-term aging is heated in an oven at 85 ± 3 °C for the corresponding length of time, with a total of five aging levels: no aging, short term, 2 d medium term, 5 d long term and 8 d ultra-long-term. The dry–wet cycle is to soak the specimen in water at 60 °C for 12 h and dry it in an oven at 30 °C for 12 h, with 24 h as a soaking cycle, and this method is used to carry out dry–wet cycles 0 times, 7 times, 14 times, and 21 times. For the actual use period of the pavement, the aging effect and the dry–wet cycle are combined in different ways, as shown below:

(1) Group 0, Group 1, Group 2, Group 3, and Group 4: unaging, short-term, 2 days medium-term, 5 days long-term, and 8 days ultra-long-term aging.
(2) Group 5, Group 6, and Group 7: 7, 14, and 21 dry–wet cycles.
(3) Group 8: 2 days medium-term aging + 7 dry–wet cycles.
(4) Group 9: 7 dry–wet cycles + 2 days medium-term aging.
(5) Group 10: 5 days long-term ag aging + 14 dry–wet cycles.
(6) Group 11: 14 dry–wet cycles + 5 days long-term aging.
(7) Group 12: 2 days ag aging + 7 dry–wet cycles + 3 days aging + 7 dry–wet cycles.
(8) Group 13: 7 dry–wet cycles + 2 days ag aging + 7 dry–wet cycles + 3 days aging.
(9) Group 14: 8 days ultra-long-term ag aging + 21 dry–wet cycles.
(10) Group 15: 21 dry–wet cycles + 8 days ultra-long-term aging.
(11) Group 16: 2 days aging + 7 dry–wet cycles + 3 days aging + 7 dry–wet cycles + 3 days ag aging + 7 dry–wet cycles.
(12) Group 17: 7 dry–wet cycles + 2 days aging + 7 dry–wet cycles + 3 days aging + 7 dry–wet cycles + 3 days aging.

## 4. Analysis of Test Results

### 4.1. Analysis of the Effect of Dynamic Modulus of Aged Asphalt Mixtures

(1) Effect of temperature on dynamic modulus

**Figure 5:**
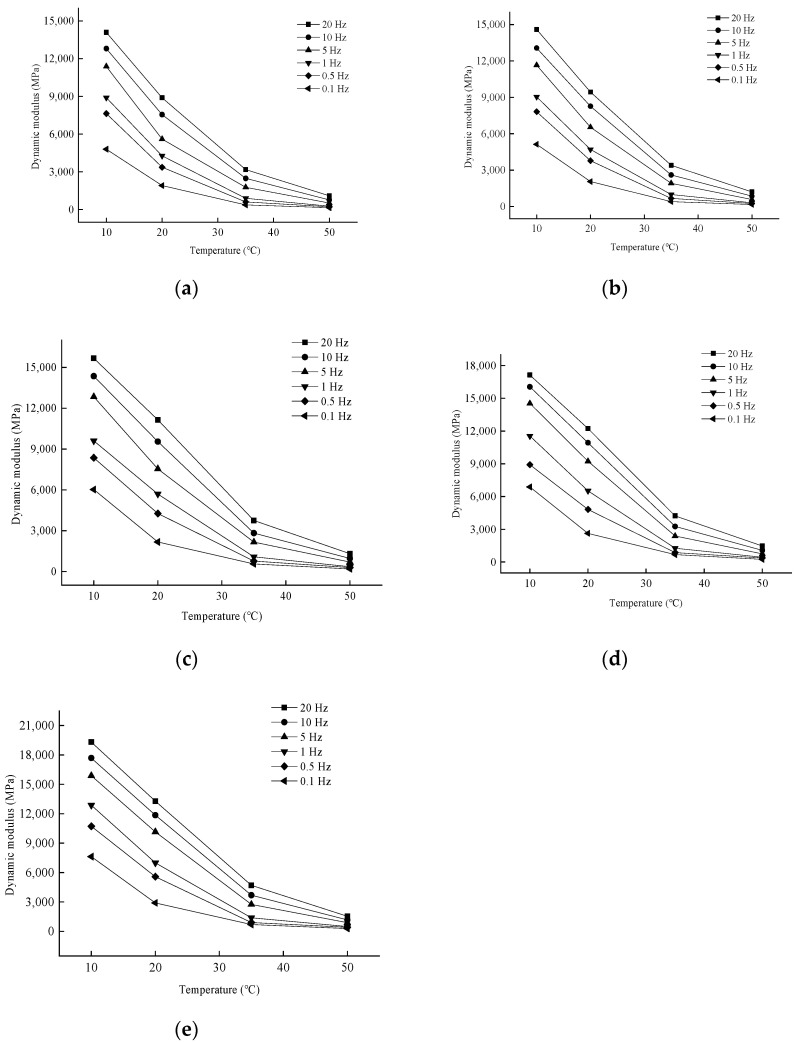
Variation of dynamic modulus with temperature for different degrees of aging. (**a**) Group 0; (**b**) Group 1; (**c**) Group 2; (**d**) Group 3; (**e**) Group 4.

In order to analyze the effect of temperature on the dynamic modulus, the variation rule of the test results is shown in Figure 5.

As shown in Figure 5, when the loading frequency is certain, the dynamic modulus under each aging degree has the same rule of change with temperature, and all of them gradually decrease with the temperature increase. This is due to the increasing temperature; the asphalt in the mixture is gradually softened, showing more viscous properties, the elastic recovery ability is reduced, and the modulus decreases.

(2) Effect of loading frequency on dynamic modulus

**Figure 6:**
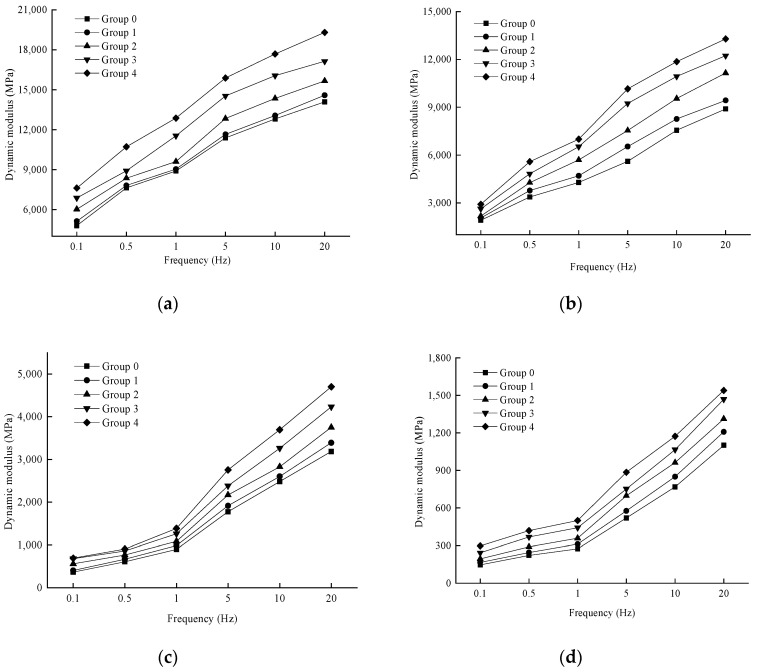
Variation of dynamic modulus with loading frequency at different temperatures. (**a**) 10 °C; (**b**) 20 °C; (**c**) 35 °C; (**d**) 50 °C.

In order to analyze the effect of loading frequency on the dynamic modulus, the variation rule of the test results is shown in Figure 6.

As shown in Figure 6, at a given temperature, the dynamic modulus of each aging degree exhibits a consistent trend with changes in loading frequency, increasing as the loading frequency rises. This behavior can be attributed to the fact that with higher loading frequencies, the mixture experiences high-frequency loading, causing the loading and unloading processes to not fully complete instantaneously, resulting in reduced strain. Additionally, due to the viscoelastic properties of asphalt mixtures, there is a certain hysteresis effect in their response to dynamic loads. As the loading frequency increases, this hysteresis phenomenon becomes more pronounced, leading to a larger performance modulus.

(3) Effect of aging on dynamic modulus

**Figure 7:**
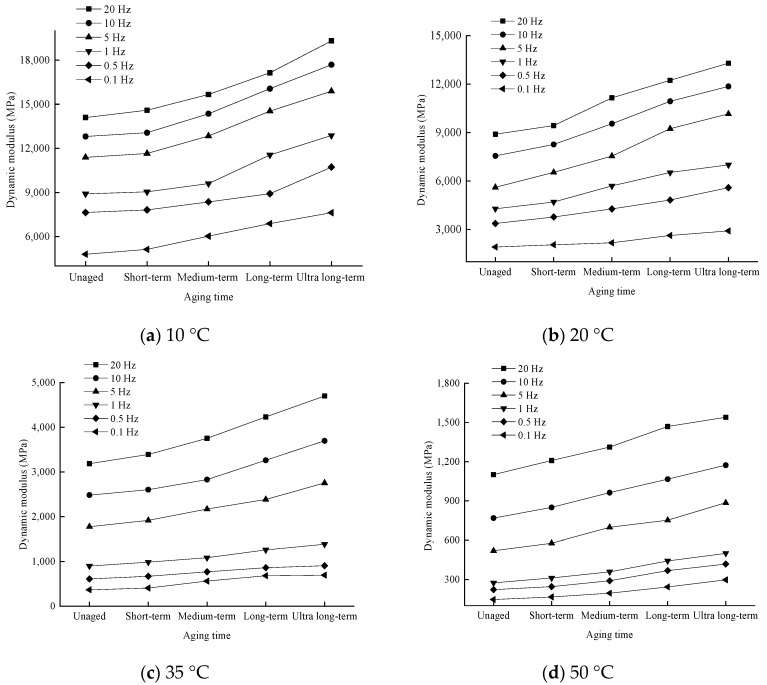
Variation of dynamic modulus with the degree of aging at different test temperatures. (**a**) 10 °C; (**b**) 20 °C; (**c**) 35 °C; (**d**) 50 °C.

In order to analyze the effect of the degree of aging on the dynamic modulus, the pattern of change in the test results is shown in Figure 7.

As seen from Figure 7, when the loading frequency is certain, the dynamic modulus at each temperature with the aging degree of the law of change is consistent, with the deepening of the degree of aging and gradual increase. At 20 °C and 10 Hz, the dynamic modulus of the unaged specimen is 7554 MPa, and the dynamic modulus of the specimen under each aging degree is 8263 MPa, 9547 MPa, 10,937 MPa, and 11,858 MPa, respectively, and the dynamic modulus is increased by 9.4%, 26.4%, 44.8%, and 57.0% compared with that of the unaged specimen.

The rise in dynamic modulus of the short-term aged specimens is relatively small, and the rise in dynamic modulus after medium-term aged and long-term aged is large. This is because, with the deepening of asphalt aging, the intermolecular forces increase dramatically, and the asphalt hardness increases, macroscopically manifested as a reduction in asphalt penetration, softening point, and viscosity increase. Bitumen and asphalt mixtures are affected by aging and gradually change to hardness and brittleness. Long-term aging also has a more pronounced hardening effect on the mix than short-term aging. The viscosity of the asphalt binder increases after aging, thus limiting the relative movement of the aggregates when the mix is subjected to external forces, and the friction between the asphalt binder and the stone material increases to a certain extent, making the mix harder and more brittle, with the uniaxial loading producing a decrease in deformation of the specimen and a significant increase in modulus.

### 4.2. Analysis of the Effect of Dynamic Modulus of Asphalt Mixtures after Dry–Wet Cycling

(1) Effect of temperature on dynamic modulus

**Figure 8:**
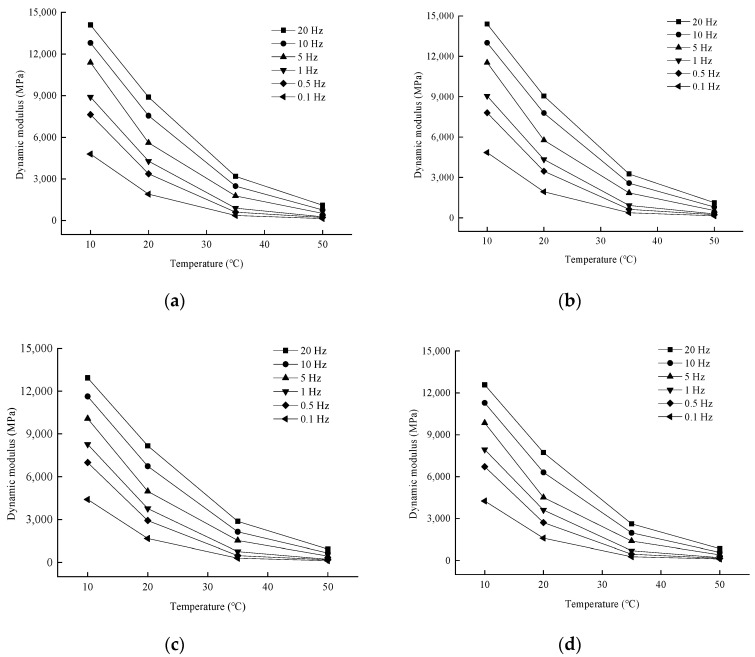
Variation of dynamic modulus with temperature for different numbers of wet and dry cycles. (**a**) No dry–wet cycle; (**b**) Seven dry–wet cycles; (**c**) Fourteen dry–wet cycles; (**d**) Twenty-one dry–wet cycles.

In order to analyze the effect of temperature on the dynamic modulus, the variation rule of the test results is shown in Figure 8.

As can be seen from Figure 8, when the loading frequency is certain, the trend of dynamic modulus and temperature change under the number of dry and wet cycles is consistent, both with the increase in temperature and decrease. When the asphalt mixture is in a high-temperature state, showing strong viscous properties, asphalt binder bonding ability by the high temperature and greatly weakened, easy to produce viscous flow, resulting in a reduction in the bearing capacity of asphalt mixtures, the deformation generated under the action of the load gradually increased, manifested as a reduction in dynamic modulus. When the asphalt mixture is in the low-temperature state, the performance of the strong elastic properties and the bearing capacity of asphalt mixtures have been enhanced, the deformation produced under the action of the load is smaller, and the performance of the dynamic modulus increases at low temperatures.

(2) Effect of loading frequency on dynamic modulus

**Figure 9:**
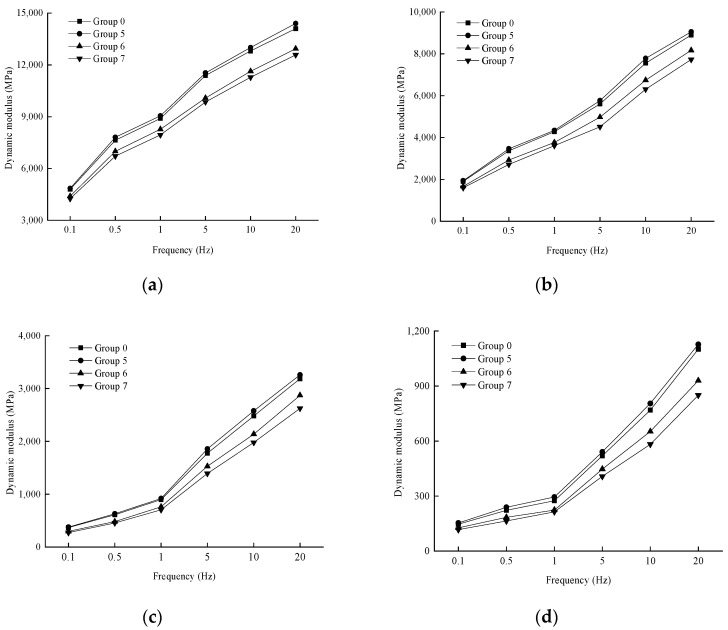
Variation of dynamic modulus with loading frequency at different temperatures. (**a**) 10 °C; (**b**) 20 °C; (**c**) 35 °C; (**d**) 50 °C.

In order to analyze the effect of loading frequency on the dynamic modulus, the variation rule of the test results is shown in Figure 9.

As can be seen from Figure 9, when the temperature is certain, the dynamic modulus under each number of dry and wet cycles and the trend of the loading frequency remains consistent with the increase of the loading frequency and increase. At the same temperature, the greater the loading frequency, the shorter the loading time; the asphalt mixture is subjected to the deformation response time generated by the load is also shorter, the response to generate a lower strain, which is manifested in the dynamic modulus increase.

(3) Effect of number of dry–wet cycles on dynamic modulus

**Figure 10:**
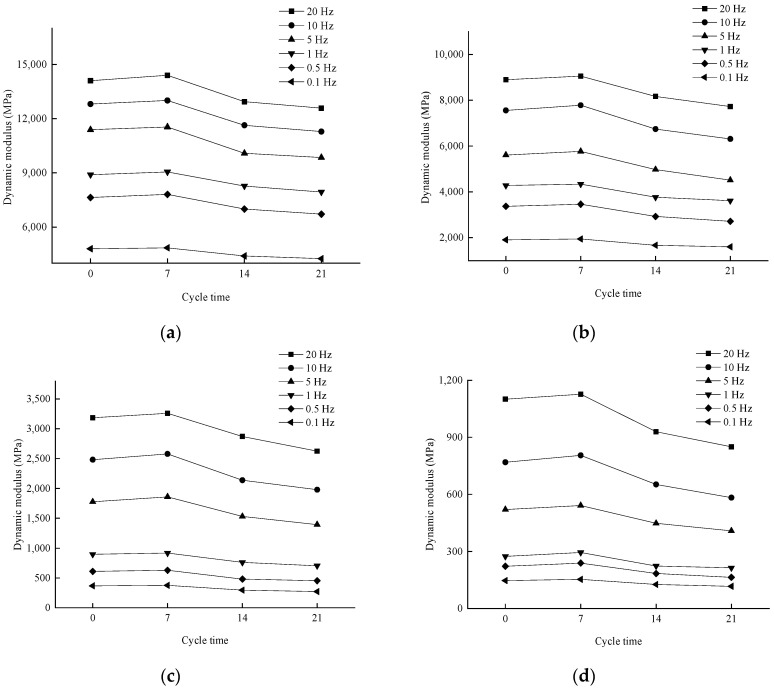
Variation of dynamic modulus with the number of dry–wet cycles at different test temperatures. (**a**) 10 °C; (**b**) 20 °C; (**c**) 35 °C; (**d**) 50 °C.

In order to analyze the effect of the number of dry–wet cycles on the dynamic modulus, the pattern of change in the test results is shown in Figure 10.

From Figure 10, it can be seen that when the loading frequency is certain, the dynamic modulus at all temperatures tends to increase and then decrease with the increase of the number of dry–wet cycles. This may be due to the fact that during the first dry–wet cycle, it is difficult for the moisture to cause a serious erosion effect on the interface between asphalt and aggregate, which has less influence on the overall stability of the asphalt mixture. While the asphalt mixture is in the process of a high-temperature water bath, the asphalt undergoes component migration, and the water aging effect is relatively more significant. During the drying process, the asphalt mixture is in full contact with oxygen, and oxidation reactions may occur. Therefore, when the asphalt mixture is subjected to 7 dry–wet cycles, the aging effect of the asphalt is obvious, and its effect is greater than the erosive effect of water on the asphalt-aggregate interface, which in general shows a slight increase in the dynamic modulus due to the aging of the asphalt occurring. When the asphalt mixture was subjected to 14 and 21 dry–wet cycles, the erosion effect of water on the asphalt mixture increased with the extension of the immersion time, the water aging effect of asphalt was gradually reduced, and the overall performance of the asphalt and the stone material due to the deterioration of the adhesion performance, the overall bearing capacity and stability, resulting in the reduction of dynamic modulus with the increase in the number of dry–wet cycles.

### 4.3. Effect of Aging and Coupled Dry–Wet Cycling on the Dynamic Modulus of Asphalt Mixtures

(1) Effects of medium-term aging and coupled dry–wet cycling on dynamic modulus

**Figure 11:**
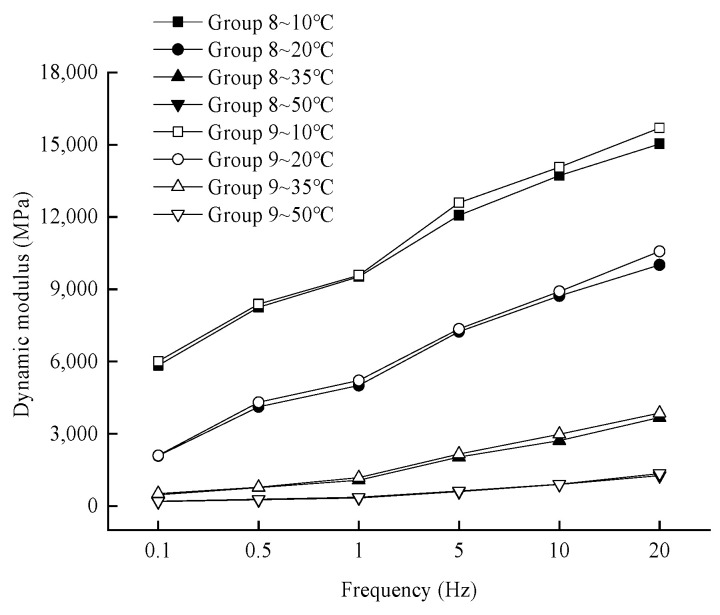
Variation of dynamic modulus with loading frequency for Group 8 and Group 9.

In order to analyze the effect of loading frequency on the dynamic modulus under different working conditions and different temperatures, the change rule of the test results is shown in Figure 11.

From Figure 11, it can be seen that the dynamic modulus variation curve with the loading frequency of Group 9 is always located above that of Group 8 at a certain temperature. Under the combination of 2-day medium-term aging and 7-day dry–wet cycles, the sequence has a certain effect on the magnitude of dynamic modulus. The dynamic modulus of the asphalt mixture with seven dry–wet cycles followed by 2-day interim aging is relatively larger. This is due to the fact that the asphalt mixture of Group 9 is subjected to dry–wet cycle first. The asphalt mixture may resist water erosion in the previous dry–wet cycle to a certain extent, and the erosion effect of water on the interface between asphalt and aggregate is weakened. Group 8, due to the first 2 days of thermo-oxidative aging, the thermo-oxidative aging leads to the asphalt component from the light component to the reorganization of the component shift; the first thermo-oxidative aging inhibits the water aging of asphalt, and at this time, the water aging effect is not obvious. The water aging effect of asphalt during dry–wet cycling is greatly weakened, and the corresponding water erosion of the asphalt-aggregate interface is more obvious. Therefore, under the conditions of 2-day medium-term aging and 7-day dry–wet cycling, the aging degree of the asphalt mixture is higher when it is first subjected to dry–wet cycling and then to thermo-oxidative aging, which is reflected in the higher dynamic modulus.

(2) Effects of long-term aging and coupled dry–wet cycling on dynamic modulus

**Figure 12:**
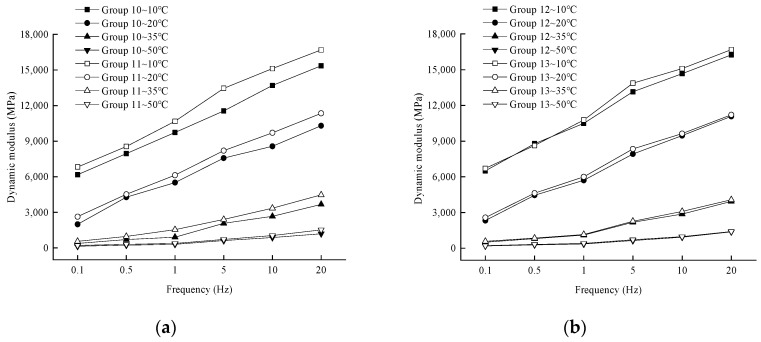
Variation of dynamic modulus with loading frequency for Groups 10, 11, 12, and 13. (**a**) Group 10, Group 11; (**b**) Group 12, Group 13.

In order to analyze the effect of loading frequency on the dynamic modulus in different groups and at different temperatures, the variation rule of the test results is shown in Figure 12.

Figure 12 shows that when the temperature is certain, the dynamic modulus variation curve with the loading frequency of Group 11 is always located above Group 10, and the dynamic modulus variation curve with the loading frequency of Group 13 is always located above Group 12. Under the direct and cross combination of 5-day long-term aging and 14 dry–wet cycles, the sequence of environmental effects has a certain effect on the dynamic modulus. Although there is a consistent pattern with the modulus change in the medium-term conditions, the reasons for the modulus change differ due to the difference in the degree of aging and the intensity of water erosion on the asphalt aggregate.

In the case of a direct combination of aging and dry–wet cycling, the water damage to the interior of the asphalt mixtures was further enhanced by the ability of water to erode the asphalt mixtures as the asphalt mixtures were subjected to 14 wet-dry cycles after long-term aging. The asphalt mixture was first subjected to 14 dry–wet cycles, and although the erosion intensity of water on the interior of the specimen was larger, the degree of water erosion and water damage was smaller compared to the former, and the voids inside the specimen were thus increased. In the asphalt mixture specimen, the internal structure is dense, and the voids are not connected to each other. As a result, the asphalt mixture internally finds it difficult to contact oxygen, and thermo-oxidative aging just relies on the asphalt to transfer, resulting in a much smaller degree of internal aging [23,24,25]. In the process of dry–wet cycle of asphalt mixture, the intrusion and evaporation of water inside the specimen, the migration of water leads to an increase in the void inside the specimen, oxygen is more likely to enter the specimen, the thermal and oxygen aging inside the specimen is more serious, and the aging of the whole specimen is more uniform. Compared with this, the asphalt mixture with 14 dry–wet cycles followed by 5 days of thermal-oxidative aging has a more significant degree of aging, which is manifested by a larger dynamic modulus at the same loading frequency and test temperature.

In the case where aging and dry–wet cycling intersect, the fact that the asphalt mixture undergoes two thermo-oxidative aging and two dry–wet cycles, in addition to the short-term aging, results in a different degree of impact on the asphalt mixture in each of the thermo-oxidative aging and water erosion. In short, the interfacial adhesion is gradually reduced by the aging effect. The two dry–wet cycles of the Group 12 specimen were after thermo-oxidative aging, resulting in a significant decrease in asphalt-aggregate adhesion and a significant increase in the degree of water damage to the mix. On the other hand, the dry–wet cycles of Group 13 specimens were both before thermo-oxidative aging, i.e., whenever the dry–wet cycles were performed, the adhesion strength at the asphalt-aggregate interface was higher in Group 13 compared to the asphalt mixture of Group 12, and the adhesion of Group 13 decreased slowly with the same number of dry–wet cycles. During the dry–wet cycling process, the moisture intruding into the asphalt and asphalt mixture does not dry out completely, and the moisture plays an exacerbating role in promoting thermo-oxidative aging, making the aging more significant in Group 13 than in Group 12. Therefore, under the effect of thermo-oxidative aging and water erosion, the aging degree of Group 13 and the adhesion effect of asphalt and aggregate are higher than that of Group 12, which is reflected in higher overall stability and higher dynamic modulus.

When the aging time and the number of dry–wet cycles is certain, the direct combination and cross combination between the aging and dry–wet cycle due to the number and order of the action of the inconsistent, each time the effect is based on the state of the specimen before the action, which appears due to the aging and water erosion on asphalt mixtures have a superimposed effect on the impact of the degree of influence of each time, there will be a certain degree of difference and uncertainty.

(3) Effects of ultra-long-term aging and coupled dry–wet cycling on dynamic modulus

**Figure 13:**
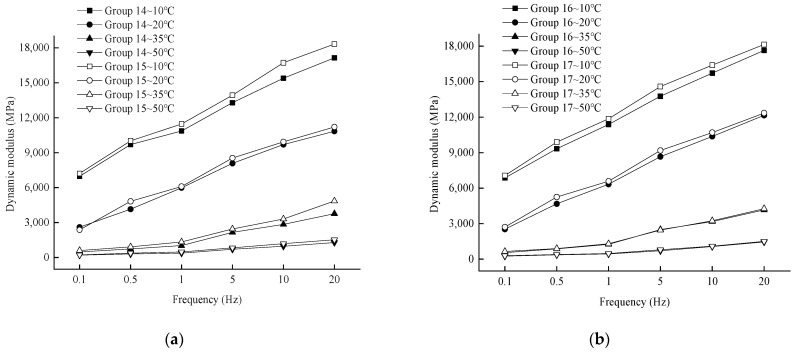
Variation of dynamic modulus with loading frequency for Groups 14, 15, 16, and 17. (**a**) Group 14, Group 15; (**b**) Group 16, Group 17.

In order to analyze the effect of loading frequency on the dynamic modulus in different Groups and at different temperatures, the variation rule of the test results is shown in Figure 13.

It can be seen from Figure 13 that the dynamic modulus of Group 15 is greater than that of Group 14, and that of Group 17 is greater than that of Group 16 at specific loading frequencies and temperatures, a phenomenon that is consistent with the trend of modulus change under long-term aging conditions. Comparing Groups 14 and 15, the ultra-long-term aging experienced by Group 14 produced a significant aging effect on the asphalt, resulting in an increased water erosion capacity during the immersion process and a detachment of the asphalt film on the aggregate surface, which in turn reduced the load-bearing capacity and overall stability of the specimens. Group 15, on the other hand, was first subjected to dry and wet cycling, and although the scouring effect of water on the interface also led to the detachment of the asphalt film, the extent of which was significantly smaller than that of Group 14. The prolonged dry and wet cycling effect caused water and gas migration inside the specimen, leading to internal pore connectivity and increased void ratio. At the same time, oxygen aggravated the internal aging of the asphalt mixture through the connected voids, increasing the homogeneity of sample aging. Under the long-term effect of moisture and thermal-oxygen aging, after 21 days of dry and wet cycling, followed by 8 days of thermal-oxygen aging, the asphalt mixtures had a higher overall load-bearing capacity, which was manifested by a higher dynamic modulus under the same conditions.

Groups 16 and 17 were divided on the basis of Groups 12 and 13 in the order of thermo-oxidative aging and wet-dry cycling. When comparing Groups 16 and 17, the fact that Group 16 underwent thermo-oxidative aging first resulted in greater subsequent water erosion and more severe damage to the asphalt-aggregate interface. In contrast, Group 17 underwent wet and dry cycling first, and the erosive effect of water on the asphalt and asphalt mixture was relatively weak. The aging of the mixes further increased under the combined effect of water, oxygen, and high temperature, and the aging effect was more intense in Group 16. The degree of aging, asphalt-aggregate interface strength, and dynamic modulus were relatively higher in Group 17 under the same frequency and temperature conditions.

## 5. Effect of Hot and Humid Environment on the Dynamic Modulus Master Curve of Asphalt Mixtures

The sigmoidal function was used to fit the dynamic modulus parameters without using temperature, circumferential pressure, and loading frequency, and the dynamic modulus master curve was obtained, as shown in Equation (2).

(2) $$\mathrm{lg}\left|E^{*}\right|=\delta +\frac{\alpha }{1+e^{\beta +\gamma \left(\mathrm{lg}t_{r}\right)}}$$

where $\left|E^{*}\right|$ is the dynamic modulus of the asphalt mixture, psi (1 MPa = 145 psi); δ is the logarithm of the minimum value of the dynamic modulus, psi; α+β is the logarithm of the maximum value of the dynamic modulus, psi; $t_{r}$ is the reduction time at the base temperature, s; β, γ is the parameter that describes the shape of the curve of the sigmoid function model.

### 5.1. Effect of Aging, and Dry and Wet Cycles on the Dynamic Modulus Master Curve

**Figure 14:**
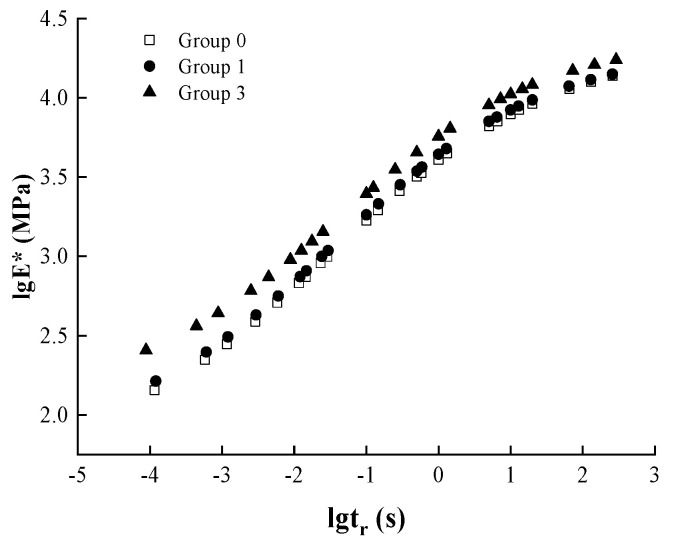
Dynamic modulus master curves for different degrees of aging.

**Figure 15:**
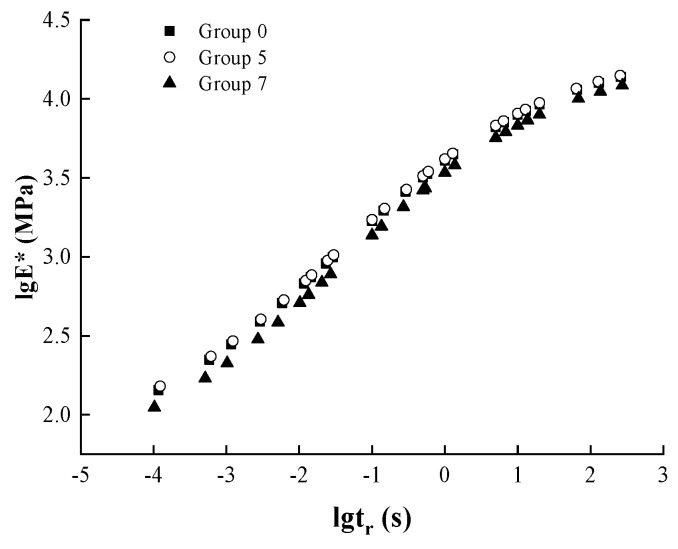
Dynamic modulus of resilience master curve for the different number of dry–wet cycles.

In order to analyze the effect of different aging levels and the different number of dry–wet cycles on the dynamic modulus master curve of asphalt mixtures, the fitting results are shown in Figure 14 and Figure 15.

As can be seen from Figure 14, the main curves of dynamic modulus under each degree of aging show “S” shape. With the increase in loading frequency, the dynamic modulus growth rate of the low-frequency section is slow, the dynamic modulus growth rate of the middle-frequency section is obvious, and the dynamic modulus growth rate of the high-frequency section slows down gradually. In the low-frequency part, according to the principle of time–temperature equivalence, low-frequency—that is, high-temperature conditions, at this time, show the asphalt viscous nature of the performance to be obvious, the dynamic modulus decreases, and the ability to resist deformation decreases.

With the deepening of the aging degree, the hardness of the asphalt mixture increases; in the whole frequency range, the main curve of the dynamic modulus gradually shifts upward—that is, the dynamic modulus increases. In the low-frequency part, i.e., under high-temperature conditions, the high-temperature rutting resistance of the mix is improved. In the high-frequency part, i.e., under low-temperature conditions, the mix is more prone to cracking, and the low-temperature rutting resistance is reduced. In this case, the degree of difference between the dynamic modulus master curve of the as-received specimen and the dynamic modulus master curve after short-term aging is small, and the difference with the modulus master curve after long-term aging is large, which further indicates that the effect of short-term aging on dynamic modulus is relatively small and that the effect of long-term aging on dynamic modulus is large.

As can be seen from Figure 15, the main curves of dynamic modulus under each number of dry–wet cycles show “S” shape. With the increase in the number of dry–wet cycles, the dynamic modulus master curve first shifts slightly upward and then decreases as a whole. This is because in the pre-process of dry–wet cycle, asphalt is easy to water aging, asphalt components change, and the asphalt gradually becomes hard. The ability to resist deformation increases at high temperatures, and at low temperatures, the stiffness modulus of the asphalt increases and shows more brittleness, decreasing crack resistance. Due to the asphalt mixture, water erosion has a certain time lag, and in the dry–wet cycle of the pre-process, the water will not immediately penetrate the asphalt membrane; at this time, the asphalt water aging effect is more obvious, the aging effect is relatively more prominent. Water aging, to a certain extent, can improve the mixture’s high-temperature performance, reducing its low-temperature crack resistance. With the increase in the number of dry–wet cycles, water on the asphalt and aggregate adhesion of the erosion increased, relative to the degree of water erosion of asphalt mixture, asphalt water aging tends to slow down, and the degree of influence is gradually reduced, the water erosion effect is more prominent. Therefore, in the late dry–wet cycle, water on the adhesion of the mixture caused serious erosion, the strength and stability of the whole structure to a certain extent, resulting in the overall weakening of the high and low-temperature performance.

### 5.2. Effect of Aging and Coupled Dry–Wet Cycling on the Dynamic Modulus Master Curve

**Figure 16:**
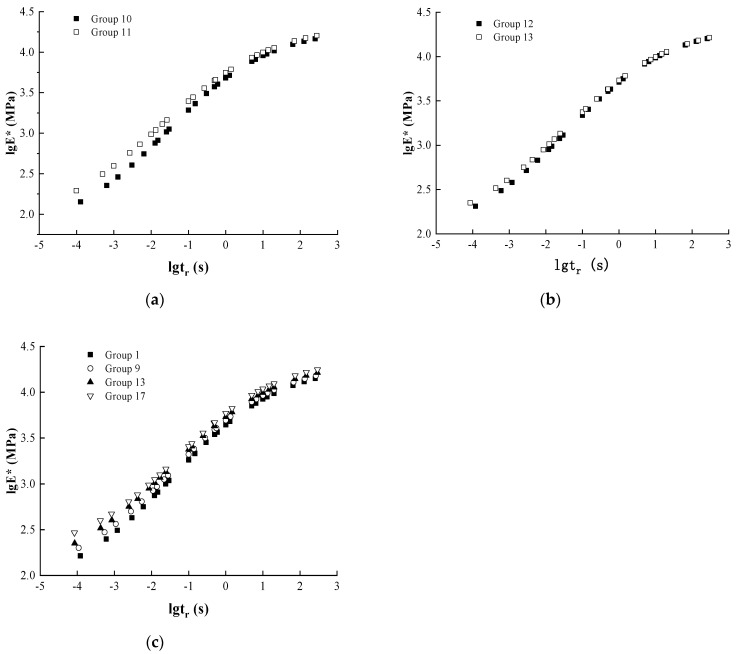
Dynamic modulus master curves for Group 1, 9, 10, 11, 12, 13, and 17. (**a**) Group 10, Group 11; (**b**) Group 12, Group 13 (**c**) Group 1, Group 9, Group 13, Group 17.

Different groups and combinations were selected as the research objects to illustrate their differences in different combinations of dynamic modulus principal curves, and are shown in Figure 16.

As can be seen from Figure 16a, the dynamic modulus master curve of Group 11 is higher than that of Group 10, which may be due to the decrease in the adhesion strength of the asphalt-aggregate interface in the asphalt mixtures after 5 days of long-term aging first, and the increase in the erosion of the water on the adhesion interface of the asphalt and aggregate in the 14-day dry–wet cycle, which reduces the stability of the whole mixture structure, resulting in the overall downward shift of the dynamic modulus master curve. The difference in the dynamic modulus master curve is larger at low frequencies and smaller at high frequencies, indicating that the sequence of aging and wet/dry cycles has a greater effect on the high-temperature performance of asphalt concrete and a smaller effect on the low-temperature performance.

As can be seen from Figure 16b, the number of aging and wet–dry cycles in the cross combination has a smaller effect on the dynamic modulus master curve. Under the cross-combination of aging and dry–wet cycles, the asphalt mixtures were subjected to inconsistent degrees of thermo-oxidative aging and water erosion, and the effects on the asphalt mixtures were different, which were related to the order of their aging and dry–wet cycle effects. Comparatively speaking, the strength of the asphalt-aggregate interface in Group 13 is more stable, and the aging effect is more significant, while the aging effect in Group 12 promotes the erosion of the interface by water, the asphalt film is more prone to peeling off, and the strength and stability of the whole specimen is weakened to a certain extent. This leads to the main curve of Group 13 being slightly higher than that of Group 12, which is less different due to the cross-interaction of aging and wet and dry cycles.

As seen in Figure 16c, the main curve of asphalt mixtures gradually shifted upward with the increase of the dry–wet cycles-aging. When the specimen is subjected to thermo-oxidative aging, the hardness increases significantly, and the modulus is elevated significantly, while when subjected to dry–wet cycling, the specimen causes water damage, and the bearing capacity of the mix decreases significantly due to the reduction of adhesion, which is manifested in the overall reduction of modulus. Although there is variability in the degree of impact due to the sequence of aging and dry–wet cycling actions, the overall trend remains that aging has a significantly higher impact on asphalt mixtures than dry–wet cycling in the combined action of a single dry–wet cycle and aging. Finally, it reflects enhanced deformation resistance at high temperatures and reduced cracking resistance at low temperatures.

## 6. Conclusions

The effects of aging, dry–wet cycling, and coupled aging and dry–wet cycling on the dynamic modulus and the principal curves of asphalt mixtures were investigated. The experimental results show that:

(1) The asphalt mixture under the erosion of hot and humid environments is still viscoelastic, the dynamic modulus decreases with the decrease of loading frequency and the increase in temperature, and its main curve is obviously an “S” type.
(2) With aging, asphalt mixture hardening effect is significant, with the deepening of the degree of aging, the dynamic modulus was an upward trend, and the dynamic modulus of short-term aged, medium-term aged, long-term aged, and ultra-long-term aged asphalt mixtures increased by 9.3%, 26.4%, 44.8%, and 57%, respectively, compared to unaged asphalt mixtures at 20 °C and 10 Hz, the mixture of high temperature rutting resistance increased, while the low-temperature crack resistance decreased.
(3) For the dry–wet cycle, the first asphalt water aging effect is more obvious, and the dynamic modulus of the mixture slightly increased. In the process of a long-term dry–wet cycle, the erosion effect of water on asphalt and aggregate increases, the structural bearing capacity attenuation, and the dynamic modulus is greatly reduced, at 20 °C and 10 Hz, for example, the dynamic modulus of asphalt mixtures with seven cycles of wet and dry cycles increased by 3% compared to asphalt mixtures without wet and dry cycles, and the dynamic modulus of asphalt mixtures with 14 cycles of wet and dry cycles and 21 cycles of wet and dry cycles decreased by 10.8% and 16.5% compared to asphalt mixtures without wet and dry cycles respectively, the main curve as a whole shift downward, and the high-temperature performance decreases significantly.
(4) In the aging and dry–wet cycle coupling, the aging asphalt mixture is more susceptible to water erosion, while the first dry–wet cycle after the mix is a relatively small degree of water erosion, the dynamic modulus is relatively larger, the high-temperature performance is relatively better, the low-temperature performance is poor.
(5) In this paper, the dynamic modulus master curves of asphalt mixtures under different operating conditions are investigated to provide a basis for predicting the dynamic modulus of asphalt mixtures under the combined effects of aging, dry and wet cycling, and extreme temperatures. The aging test was carried out using only the thermo-oxidative aging method, which can be further improved by combining with the UV aging method. The asphalt was extracted and analyzed from the perspective of asphalt. It is recommended that the aging mechanism be elaborated on under humid and hot environments with the help of asphalt microtests and that the aging state of the pavement core samples be reasonably assessed in the course of subsequent research. At the same time, new indicators and methods are used to evaluate the mechanical properties of asphalt mixtures.

## Figures and Tables

**Table 1:** Technical specifications and test results of No. 70 matrix asphalt.

Test Item		Unit	Technical Requirements	Test Results	Test Methods
Needle penetration 25 °C		0.1 mm	60~80	67	T 0604-2011 [19]
Needle penetration index PI		-	−1.5~+1.0	−1.34	T 0604-2011
Ductility(10 °C, 5 cm/min)		cm	≥20	27.5	T 0605-2011
Ductility(15 °C, 5 cm/min)		cm	≥100	150	T 0605-2011
Softening point		°C	≥47	47.3	T 0606-2011
Power viscosity 60 °C		Pa·s	≥180	190	T 0620-2011
Flash point		°C	≥260	>260	T 0611-2011
Solubility (trichloroethylene)		%	≥99.5	>99.5	T 0607-2011
After heating the film	Mass change	%	−0.8~+0.8	0.03	T 0609-2011
	Needle penetration ratio 25 °C	%	≥61	65	T 0604-2011
	Ductility 10 °C	cm	≥6	6.3	T 0605-2011
Density at 25 °C		g/cm^3^	-	1.04	T 0603-2011

**Table 2:** Technical specifications and test results of coarse aggregate.

Test Item	Unit	Technical Requirements	Test Results	Test Methods
Crushing value	%	≤20	13.8	T 0316-2005 [20]
Los Angeles abrasion losses	%	≤24	7.4	T 0317-2005
Grinding value	PSV	≥42	48	T 0321-2005
Apparent relative density	g/cm^3^	≥2.6	2.920	T 0304-2005
Water absorption	%	≤2.0	0.2	T 0304-2005
solidity	%	≤12	1	T 0314-2005
Content of needle and flake particles	%	≤15	2.0	T 0312-2005
Mud content (<0.075 mm content)	%	≤1	0.2	T 0333-2005

**Table 3:** Fine aggregate technical specifications and test results.

Test Item	Unit	Technical Requirements	Test Results	Test Methods
Apparent relative density	g/cm^3^	≥2.5	2.728	T 0328-2005
Sand equivalent	%	≥65	78	T 0334-2005
Angularity	s	≥30	40	T 0345-2005

**Table 4:** Mineral powder technical specifications and test results.

Test Item		Unit	Technical Requirements	Test Results	Test Methods
Apparent relative density		g/cm^3^	≥2.5	2.729	T 0352-2005
Hydrophilicity coefficient		%	<1	0.71	T 0353-2005
Heating stability		-	Measurement records	No colour change	T 0355-2005
Exterior appearance		-	No agglomeration	No agglomeration	-
Particle size range (%)	<0.6 mm	%	100	100	T 0351-2005
	<0.15 mm	%	90~100	99.4	T 0312-2005
	<0.075 mm	%	75~100	88.9	T 0616-2005

**Table 5:** AC-13C proportion validation and technical specifications.

Gradation	Gross Bulk Density	Void Ratio	Bitumen Saturation	Stability	Flow Value	Mineral Gap Ratio
	(g/cm^3^)	(%)	(%)	(kN)	(kN)	(%)
AC-13C	2.527	4.3	70.7	13.42	2.526	14.6
technology Specifications	-	4~6	65~75	≥8	1.5~4	≥14

**Table 6:** AC-13C performance verification and technical specifications.

Gradation	Dynamic Stability (Times/min)	Soaked Marshall Stability MS_0_ (%)	Freeze–Thaw Splitting Tensile Strength TSR (%)
AC-13C	2831	82.6	82.1
Technical Specifications	≥1000	≥80	≥75

## Data Availability

The data presented in this study are available on request from the corresponding author.
